# Prenatal diagnosis of coarctation of the aorta with a long and angled isthmus by two- and three-dimensional echocardiography: a case report

**DOI:** 10.1186/s12872-021-01987-7

**Published:** 2021-04-13

**Authors:** Yu Wang, Caixia Liu, Ying Zhang, Meilian Wang

**Affiliations:** 1 Department of Ultrasound, Shengjing Hospital of China Medical University, Shenyang, Liaoning China; 2 Department of Microbiology and Parasitology, College of Basic Medical Sciences, China Medical University, Shenyang, Liaoning China

**Keywords:** Fetus, Coarctation of the aorta, 3D, Spatiotemporal image correlation

## Abstract

**Background:**

Prenatal diagnosis of coarctation of the aorta (CoA) is challenging for most examiners. The malformation often occurs at the aortic isthmus, which is a short segment between the origin of the left subclavian artery and the insertion of the ductus. We report herein a rare case of CoA with a long, angled, and hypoplastic isthmus. The echocardiographic characteristics and postmortem findings are presented to approach the skill of fetal diagnosis.

**Case presentation:**

A pregnant women undergone fetal echocardiography at 26 + 3 gestational weeks in our center. Conventional two-dimensional echocardiography (2DE) showed that ascending aorta went straight upward branching three brachiocephalic arteries without the appearance of the arch, suggesting the possibility of an interrupted aortic arch. Three-dimensional echocardiography (3DE) using spatiotemporal image correlation (STIC) and high-definition flow imaging technique was performed to obtain the 3D rendered images, which clearly showed the arch and its angled junction with the slim isthmus in space. Intra-uterine fetal death occurred and an autopsy was performed. The gross findings showed the angled hypoplastic aortic isthmus in detail and thus confirmed the prenatal diagnosis.

**Conclusions:**

Traditional 2DE may be limited in showing the angled hypoplastic aortic isthmus, while the 3DE STIC technique can provide additional spatial information to show great arteries in detail, help to find tiny vessels, and thus benefit the examiners to make an accurate diagnosis.

**Supplementary Information:**

The online version contains supplementary material available at 10.1186/s12872-021-01987-7.

## Background

The aortic isthmus is a part of the aorta locating between the origin of the left subclavian artery and the ductus. In fact, the narrowing occurs in this segment in more than 90% cases of coarctation of the aorta (CoA) [1]. Usually, the isthmus is very short, only about 1 cm in length. A discrete stenosis may present when coarctation occurs at this position. Under very rare conditions, a long, slim and hypoplastic isthmus would appear, forming the junction between the aortic arch and descending aorta. In this study, we report a fetal case of CoA with this “special” isthmus. Conventional two-dimensional echocardiography (2DE) made a diagnosis of interrupted aortic arch (IAA) as the attachment of the arch to the ductus and descending aorta was invisible. Three-dimensional echocardiography (3DE) using spatiotemporal image correlation (STIC) and high-definition flow imaging (HDFI) technology was then performed to obtain the 3D reconstructed image, which helped to reach an accurate diagnosis of CoA confirmed by postmortem findings.

## Case presentation

**Fig. 1:**
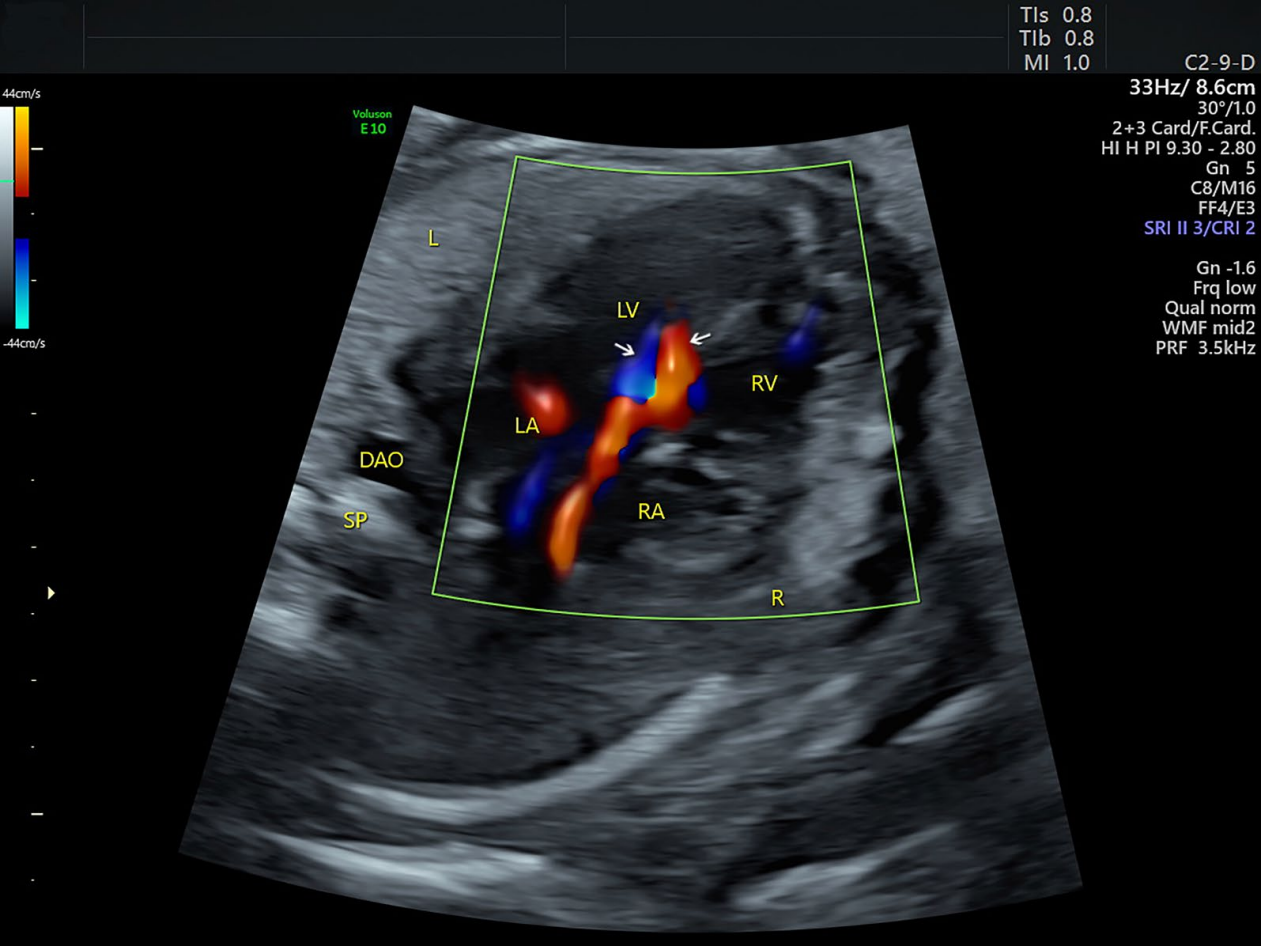
Sonograms of the fetus of coarctation of the aorta at the four-chamber view. The four-chamber view shows symmetrical left and right chambers. In addition, a bidirectional shunt (indicated by the arrows) between the left and right ventricles is demonstrated by color Doppler. DA: ductus arteriosus; DAO: descending aorta; L: left; LA: left atrium; LV: left ventricle; R: right; RA: right atrium; RV: right ventricle; SP: spine

**Fig. 2:**
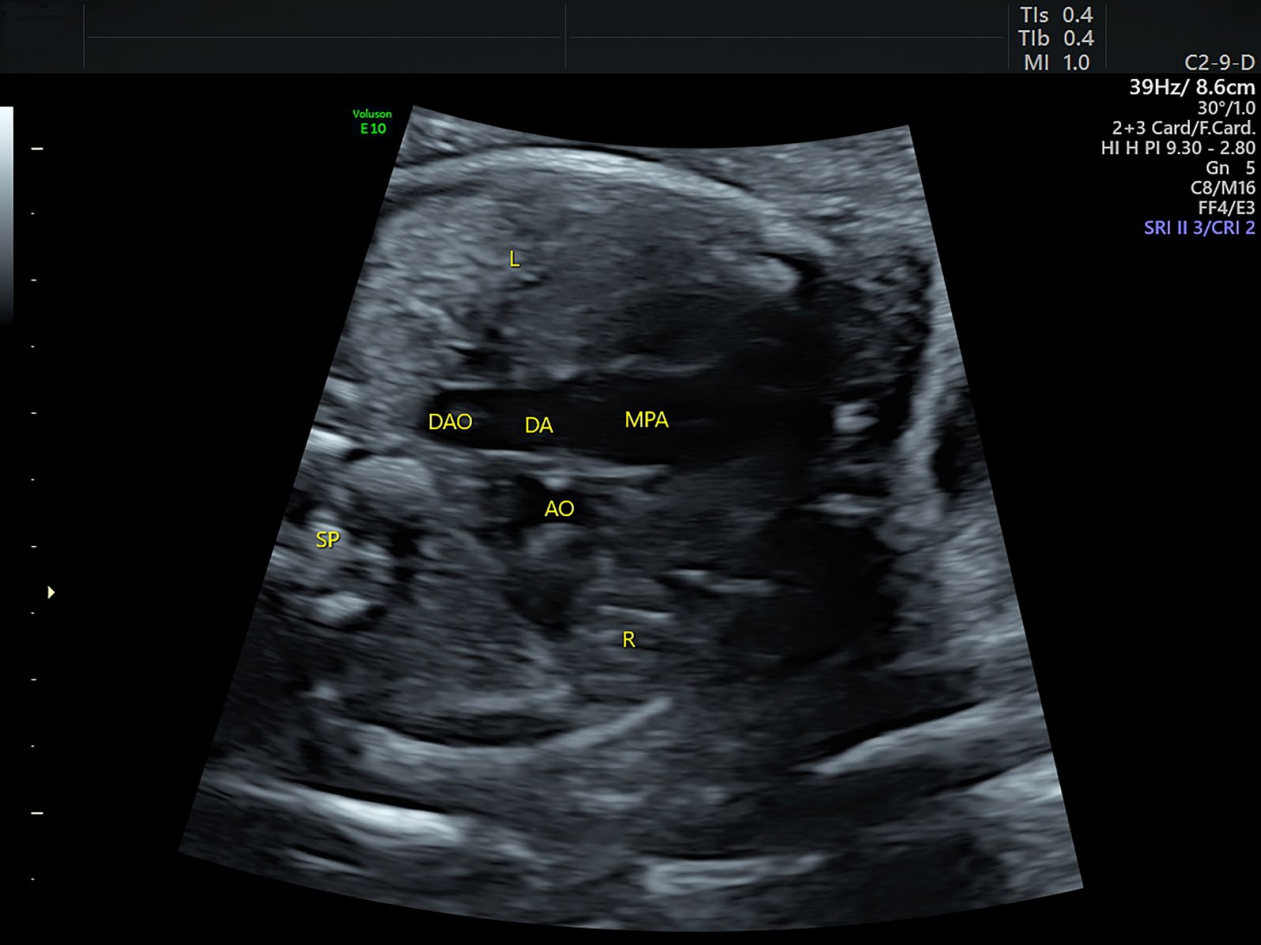
Sonograms of the fetus of coarctation of the aorta at the three-vessel trachea view. The large-sized pulmonary artery is visualized to connect with the descending aorta via the ductus. The aorta is on the right side of the pulmonary artery but is not visualized to drain into the ductus. AO: aorta; DAO: descending aorta; L: left; MPA: main pulmonary artery; R: right; SP: spine

**Fig. 3:**
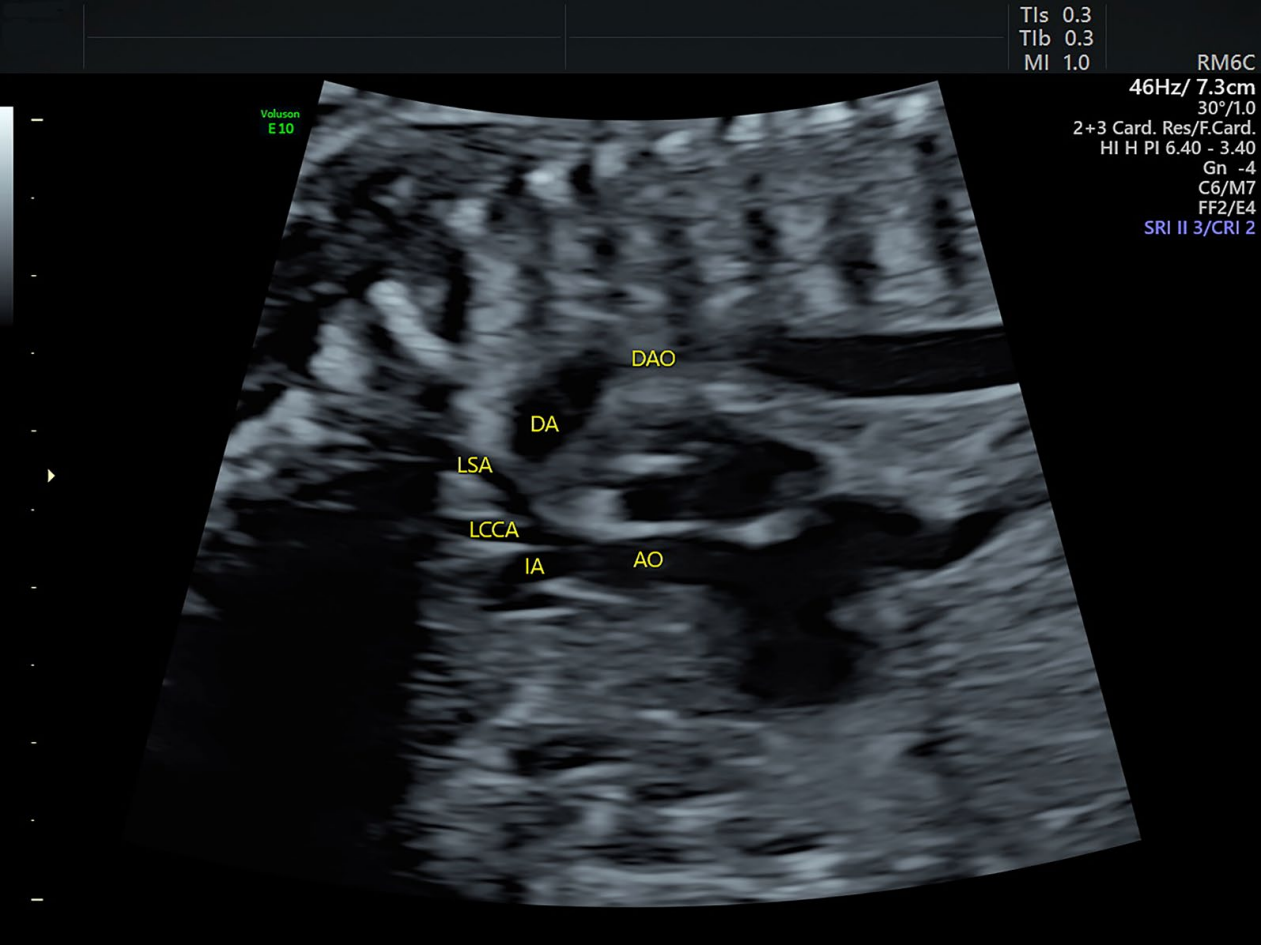
Sonograms of the fetus of coarctation of the aorta at the sagittal view. The sagittal view shows that the ascending aorta goes straight upward and gives off three brachiocephalic arteries while the aortic arch does not appear. AO: aorta; DA: ductus arteriosus; DAO: descending aorta; IA: innominate artery; LCCA: left common carotid artery; LSA: left subclavian artery

**Fig. 4:**
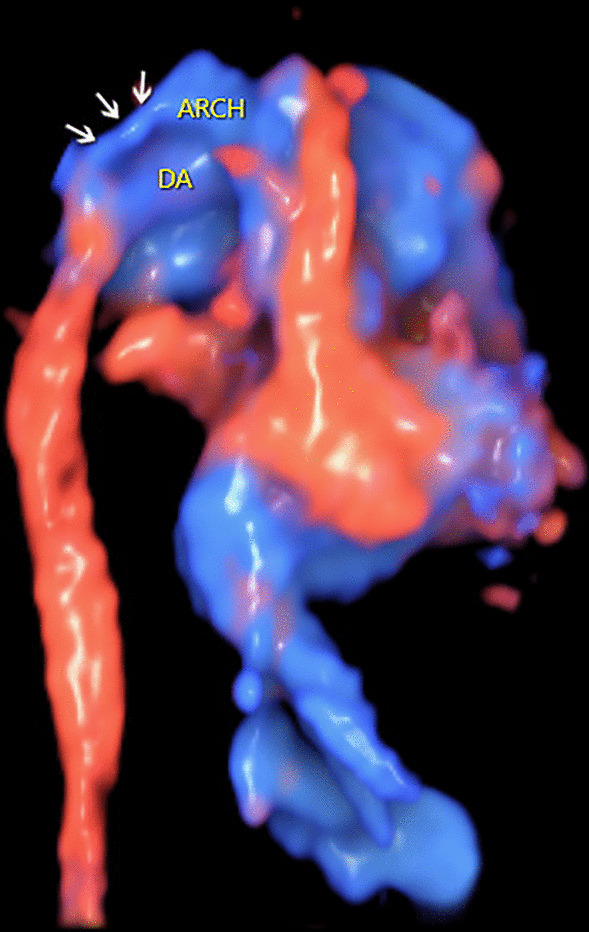
The 3D reconstructed image showing the great arteries of the fetus with coarctation of the aorta. The 3D rendered image clearly shows the great arteries in space. In addition, it demonstrates a sharp angle between the aortic arch and the slim isthmus (indicated by the arrows). Arch: aortic arch; DA: ductus arteriosus

A 32-year-old woman, gravida 3, para 1, was referred to our center at 26 + 3 weeks of gestation for further fetal cardiac examination for suspected cardiac anomalies. The patient was in good health without any maternal complications or high-risk factors (e.g. diabetes, hypertension, amniotic disorders). A detailed echocardiogram was performed to find any potential cardiac anomalies using transverse and sagittal scanning. Conventional 2DE was used to show fetal anatomical structures. Color Doppler flow imaging (CDFI), together with HDFI was used to show fetal hemodynamics. The sound beam was continuously moved upward along the vertical axis of the fetal thorax to perform the transverse scanning. The four-chamber view (4CV) showed a symmetrical left and right heart. The left and right outflow tract views showed normal ventriculoarterial connections. However, a small-sized ventricular septal defect (VSD) was identified at the 4CV and the left outflow tract view with the communication clearly shown by CDFI (Fig. 1). An additional movie file shows this in more detail (See Additional file 1: Video). The pulmonary valve annulus (PVA) was apparently wider in comparison with the aortic valve annulus (AVA). The ratio of PVA/AVA was 1.6 in diameter. The size of the pulmonary artery (PA) was also larger than the aorta (AO) and the ratio of PA/AO was 2.1 in diameter. At the three-vessel trachea (3VT) view, the widened pulmonary trunk continued to be the ductus, which was connected with the descending aorta. At the same time, a tiny vessel was found located at the right side of the pulmonary trunk, with no convergence with the ductus (Fig. 2). An additional movie file shows this in more detail (See Additional file 2: Video). Under normal conditions, the aortic arch joins with the ductus to form proximal descending aorta in a characteristic “V” shape at the 3VT view [2]. The echocardiographic manifestations in the current case thus suggest the possibility of an IAA. Sagittal planes were also scanned to obtain more information. The arch did not present, instead, the ascending aorta was visualized going straight upward and then branching three brachiocephalic arteries in a “W” shape (Fig. 3). An additional movie file shows this in more detail (See Additional file 3: Video). A preliminary diagnosis of IAA type A was then reached. The 3DE with STIC technology was then used to obtain 3D images of the great arteries to confirm the 2D diagnosis. A 3D motorized transducer (4–8 MHz) was used to acquire cardiac volumes when scanning the sagittal planes using HDFI. The acquisition time was set to 12.5 s and the sweep angle was set to 30°. Cardiac volumes were acquired automatically and then reconstructed to display in a cine loop in multiplanar mode. Volume post-analysis was then performed using an off-line software (4D viewer, version 14.0) to obtain the 3D reconstructed images. This could be achieved by properly adjusting the direction and size of the region of interest (ROI) and the rotation of the images in three orthogonal planes in the volume. A combination of smooth surface and gradient light algorithms was also used to enhance the 3D effect of the reconstructed images. The 3D image demonstrated an abnormal angle of attachment of the aortic arch to the ductus and descending aorta via a slim isthmus (Fig. 4). An additional movie file shows this in more detail (See Additional file 4: Video). The final diagnosis was CoA at the isthmus.

**Fig. 5:**
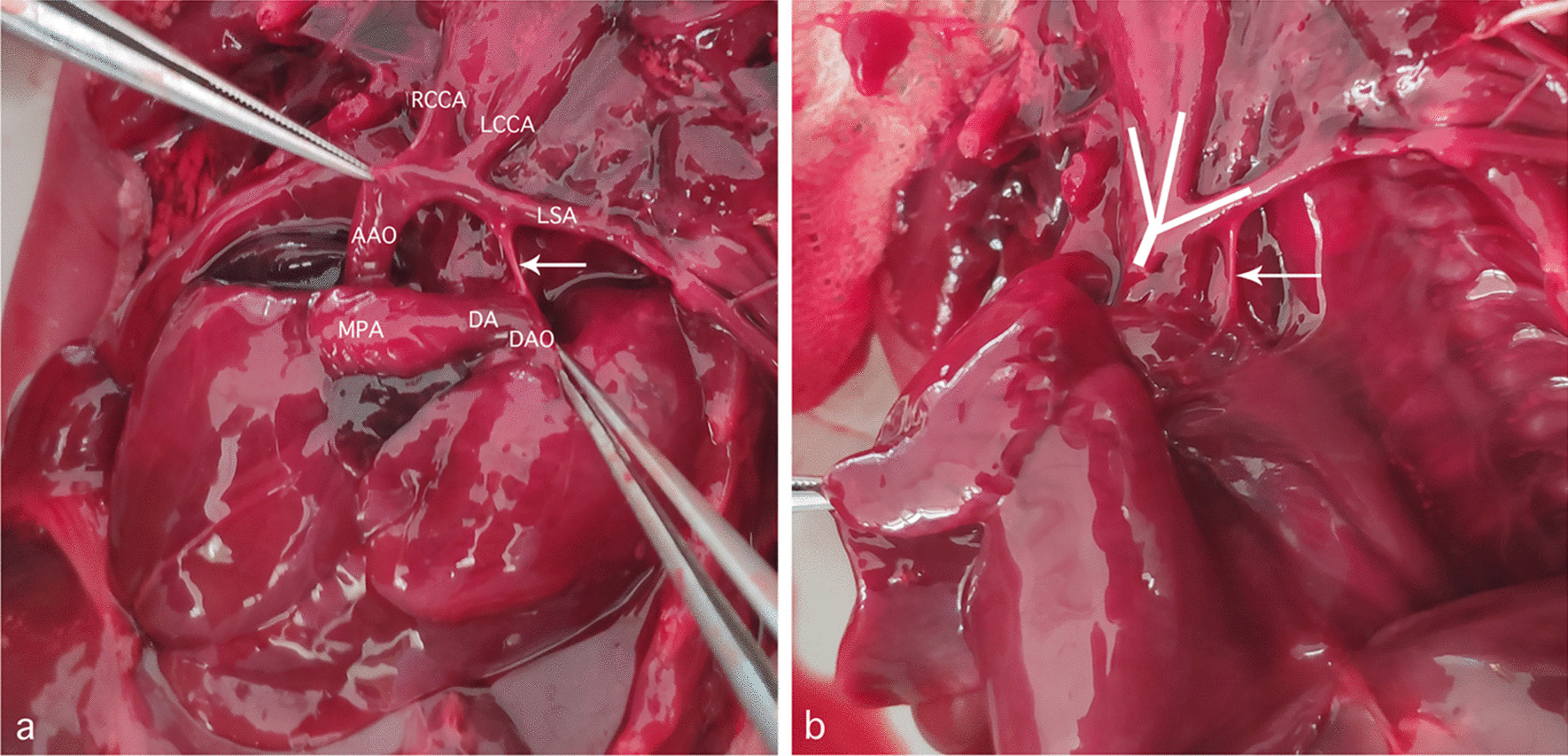
Gross findings of the fetus of coarctation of the aorta. The branching patterns of aortic arch and the slim isthmus (indicated by the arrow), together with its connection with descending aorta are clearly shown, when pulling up the great arteries using two forceps (**a**). The original position of the great arteries is also shown (**b**). Three white lines are drawn to show the aortic arch branches, which are presenting in a “W” shape. The course of the arch is not transverse but oblique upward, leading to a very sharp angle of its connection with the isthmus (indicated by the arrow)

The patient refused a chromosomal examination and did not go back to the following-up echo four weeks later for personal reasons. She came to the hospital at 31 gestational weeks as she could not feel fetal movement. Intra-uterine fetal death was demonstrated by fetal echo and proofs of intra-uterine infection were found during the induction of the labor. An autopsy was then performed and showed the great arteries in detail. The spatial relationships of the aorta and the pulmonary artery were normal. The size of the ascending aorta was approximately half of that of the pulmonary trunk while it went straight upward and gave off the innominate artery, left common carotid artery and the left subclavian artery, in turn. An abnormal angle of the connection between the aortic arch and the slim isthmus could be appreciated (Fig. 5). In addition, a perimembranous VSD about 4 mm in diameter was also found. The gross findings confirmed the prenatal diagnosis.

## Discussion

CoA is a form of deformity that stenosis presents in a segment of the aortic lumen along the arch, which may lead to an obstruction of the blood flow. It occurs in about 6–8% of live births with congenital heart disease [1]. In general, CoA can be classified into three types according to the location of the narrowing relative to the ductus. The first one is the pre-ductal coarctation in which the narrowing presents proximal to the insertion of the ductus. The second form is the ductal coarctation where the narrowing occurs at the junction of the ductus. This type of coarctation is usually associated with the prominent posterior infolding of the aortic wall (the posterior shell). The last type is the post-ductal coarctation, in which the narrowing occurs distal to the insertion of the ductus. For the three types of CoA, the pre-ductal coarctation is most common in utero.

Fetal diagnosis of CoA is generally inferred from secondary findings of ventricular and great artery disproportion [3–6]. Right-sided structures dilatation relative to the left-sided structures usually serves as the first clue to the diagnosis. Previous studies indicated a larger ratio of right ventricle/left ventricle (> 1.5) [4] and PVA/AVA (> 1.6) [5–7] in diameter for CoA fetuses, which could be assessed at the 4CV and the outflow tracts views, respectively. Note that the size discrepancy may not be obvious for bilateral ventricles in the presence of a VSD and the first sign is the great artery disproportion. Our result is consistent with this point. Aortic isthmus diameter Z scores could be measured either in the sagittal view or the 3VT view, which are significantly lower in fetuses with CoA (< 3 standard deviations than normal fetuses with the same gestational age) [7, 8]. A larger ratio (> 1.4) of ductus/aortic isthmus in diameter could also suggest the presence of CoA [4]. In addition, the larger distance between the left common carotid artery and the left subclavian artery [8] and some intracardiac complications (e.g. VSD, persistent left superior vena cava, and bicuspid aortic valve) [3] should also call attention to the possibility of CoA. Though we discuss above the needed echo parameters, capabilities, and some criteria to evaluate fetal CoA, it is really not easy to make an accurate prediction or diagnosis in utero. Wide differential diagnosis including pulmonary dilatation (e.g. pulmonary hypertension, premature ductal constriction) is required [9]. Fetal blood redistribution may also contribute to the false diagnosis of fetal CoA. Compression of fetal neck by the umbilical cord might result in a disproportion of cardiac flow, “mimicking CoA”, as reported by Więckowska et al. [10]. In addition, different hemodynamics patterns may present for fetuses and neonates when the ductus closes. It is better to be considered as a provisional diagnosis for CoA in utero, which should be either confirmed or refuted after birth as a high false-positive diagnosis is common in fetal life.

We want to stress that CoA is difficult to differentiate from IAA type A in some cases, as the site of the lesion is at the isthmus for both circumstances. For embryonic development, different degrees of regression of the left dorsal aorta at this specific segment lead to the formation of a narrowing aortic lumen, an atretic fibrous band or complete absence of that portion of the arch [11]. Slodki et al. [12] suggested that a large discrepancy between PA/AO diameters should raise the suspicion of aortic arch anomalies. In their studies, the calculated ratio of PA/AO was about 2.1 in IAA type A fetuses. Similar disproportional great arteries were found in the current COA fetus, which suggested an insufficient proof to make a differential diagnosis based only on scanning the three-vessel view. It is necessary to move the sound beam more cephalad to determine whether the ductus join with aorta at the 3VT view. The disjoint of the two great arteries suggests the possibility of an inexistent arch. However, it might also be possible that the arch and/or the isthmus is too slim to be seen in this plane in some rare conditions. Sagittal planes may be useful in showing the course and branching of the aorta. Previous report [13] indicated that the ascending aorta goes straight upward to the fetal head without the appearance of a curved arch in terms of IAA. However, we found that the course and angle of ascending aorta in current CoA fetus seem like those of IAA, which adds the difficulty to make a differentiation. In fact, the gross finding shows a long isthmus with a relatively short arch. The course of the arch is not transverse but oblique upward, leading to a very sharp angle of its connection with the isthmus, in comparison with the normal smooth junction between the adjacent structures. Based on these points, the arch was misrecognized as parts of the ascending aorta with the angled slim isthmus invisible during the sagittal scanning.

Volume sonography is the 3D technique used in fetal echocardiography [14]. It was initially proposed as a concept of research but now have been proved to provide much diagnostic information in addition to conventional 2DE. Briefly, STIC technology is used to acquire datasets containing many 2D images and then combine into a single cardiac volume [15, 16]. It has also been adapted to acquire volumes with color information, including color Doppler, HDFI, and B-flow imaging. In our previous studies, we have shown that the 3D technique helped to diagnose fetal tubular arch hypoplasia [17], IAA [13], and arch anomalies [18]. Herein, we further proved its value in the diagnosis of fetal CoA. The narrowing aortic lumen was clearly shown with blood filling, indicating a stenosis isthmus instead of an interruption. The anatomical structures and spatial relationships of the great arteries could be vividly shown in rendered HDFI images in an in-depth perspective, which resembles the autopsy findings very much. Another benefit, the addition of depth perception in the 3D reconstructed image actually shows anatomical information of multiple 2D planes, some of which could not be obtained during the routine 2D scan. We speculate it is the reason for the correct diagnosis of the 3D modality in the current report.

Previous reports had indicated that prenatal diagnosis of CoA improves survival and reduces morbidity [19, 20]. We report herein an accurate diagnosis of this malformation by 3D modality, which also ensured a better prenatal counseling for the parents, it is a benefit of our research. In addition, it is extremely rare that intrauterine death occurred for CoA fetuses, which is not reported before. We expect this “special” case of fetal CoA may benefit screening sonographers and obstetricians.

## Conclusion

We present a fetal case of CoA diagnosed by 2DE and 3DE-STIC technology. The traditional 2DE has some limitations in showing the angled and hypoplastic aortic isthmus, while the 3D rendered image may provide additional spatial information to show great arteries in detail, help to find tiny vessels, and thus benefit the examiners to make an accurate diagnosis.

## Supplementary Information

**Additional file 1**. Video. Sonograms showing symmetrical left and right ventricles and bidirectional communication between the two ventricles at the four-chamber view.

**Additional file 2**. Video. Sonograms showing the three-vessel trachea view in a fetus with coarctation of the aorta. The large-sized pulmonary artery is visualized to connect the descending aorta via the ductus. The aorta is on the right side of the pulmonary artery but is not visualized to drain into the ductus.

**Additional file 3**. Video. Sonograms showing the sagittal view in a fetus with coarctation of the aorta. It shows that the ascending aorta goes straight upward and gives off three brachiocephalic arteries while the aortic arch does not appear.

**Additional file 4**. Video. The 3D rendered image cine loops showing the great arteries in a fetus with coarctation of the aorta. The 3D rendered image clearly shows the great arteries in space. In addition, it demonstrates a sharp angle between the aortic arch and the slim isthmus (indicated by the arrows). Arch: aortic arch; DA: ductus arteriosus.

## Data Availability

The datasets supporting the conclusions of this article are included within the manuscript (and its additional files). The authors would like to share raw anonymized video data related to the current study, which could only be used for personal study. The demanders may contact baogoubei@hotmail.com.

## Abbreviations

2DE: Two-dimensional echocardiography
3DE: Three-dimensional echocardiography
3VT: Three-vessel trachea
4CV: Four-chamber view
AO: Aorta
CDFI: Color Doppler flow imaging
CoA: Coarctation of the aorta
HDFI: High-definition flow imaging
IAA: Interrupted aortic arch
PA: Pulmonary artery
STIC: Spatiotemporal image correlation
VSD: Ventricular septal defect
